# A wearable motion capture device able to detect dynamic motion of human limbs

**DOI:** 10.1038/s41467-020-19424-2

**Published:** 2020-11-05

**Authors:** Shiqiang Liu, Junchang Zhang, Yuzhong Zhang, Rong Zhu

**Affiliations:** State Key Laboratory of Precision Measurement Technology and Instrument, Department of Precision Instrument, Tsinghua University, Beijing, 100084 China

**Keywords:** Health care, Biomedical engineering

## Abstract

Limb motion capture is essential in human motion-recognition, motor-function assessment and dexterous human-robot interaction for assistive robots. Due to highly dynamic nature of limb activities, conventional inertial methods of limb motion capture suffer from serious drift and instability problems. Here, a motion capture method with integral-free velocity detection is proposed and a wearable device is developed by incorporating micro tri-axis flow sensors with micro tri-axis inertial sensors. The device allows accurate measurement of three-dimensional motion velocity, acceleration, and attitude angle of human limbs in daily activities, strenuous, and prolonged exercises. Additionally, we verify an intra-limb coordination relationship exists between thigh and shank in human walking and running, and establish a neural network model for it. Using the intra-limb coordination model, dynamic motion capture of human lower limbs including thigh and shank is tactfully implemented by a single shank-worn device, which simplifies the capture device and reduces cost. Experiments in strenuous activities and long-time running validate excellent performance and robustness of the wearable device in dynamic motion recognition and reconstruction of human limbs.

## Abstract (web summary)

Current wearable motion capture technologies are unable to accurately detect dynamic motion of human limbs due to drift and instability problems. Here, the authors report a wearable motion capture device combining tri-axis velocity sensor and inertial sensors for accurate 3D limb motion capture.

## Introduction

Motion capture technology plays an essential role in action recognition, motor function assessment and dexterous human-robot interaction for rehabilitation robots and intelligent prosthetics. It allows machine to assist users and improve life quality in such as senior care, physical rehabilitation, daily life-logging, personal fitness, and assistance for people with cognitive disorders and motor dysfunctions^1–6^. Optical system (or machine vision system) is one of the most popular solutions for motion capture. However, high cost, complex setup and susceptibility to lighting condition and occlusion limit their applications only for laboratory^7,8^. Goniometer (or angle encoder) is another commonly-used motion capture device applied in rehabilitation robotics. But its noncompliance with human joint that has multi-degree-of-freedom disturbs the natural pattern of human motion and leads to discomfort and even joint injury in long-term applications^9,10^. Wearable motion sensors, such as force based sensors^11–13^, surface electromyography sensors^14–16^, soft strain sensors^17–20^, and micro inertial sensors^21^, may overcome such problems. The wearable sensors provide promising tools for the next generation rehabilitation exoskeletons, such as soft exosuits, in which lightweight and comfort are concerned^17,22^. Among these sensors, body-worn inertial sensors including micro accelerometers and micro gyroscopes are the most commonly used wearable movement sensors^23,24^ due to their capability of direct measurement on body segment movement, which is important for not only quantitative assessment of motor function^9^, but also interaction and control of rehabilitation robots and prostheses^25^.

Although the popular uses of inertial sensors in motion capture, technique challenges still exist in detecting dynamic motion of human limbs. As a matter of fact, the accelerometer detects total acceleration of gravity and motion accelerations^26,27^ that precludes it from determining motion velocity or attitude angle independently, and gyroscope based attitude estimation suffers from integral drift error. The motion velocity is usually estimated using integral of motion acceleration. However, due to the lack of precise motion acceleration, even processed by noise filtering and fusion method, the motion acceleration determined by inertial sensors still contains noises and errors that induces the cumulative error in the integral for estimating the motion velocity. To solve the drift problem, a variety of calibration algorithms have been proposed, including the model-based method^28–30^, the machine-learning-based method^31^, the Zero Velocity Update (ZUPT) method^9,32^, and the drift estimation method^33^. However, these methods restrict only for foot-mounted or shank-mounted applications, and large acceleration interference caused by foot strike degrades the velocity tracking performance. To estimate attitude angles, various data fusion solutions using accelerometer and gyroscope have been investigated, such as acceleration threshold-based method^34,35^ and model-based method using Kalman filters or complementary filters^26,36,37^. The threshold-based method regulates the weight of the accelerometer in data fusion according to the intensity of acceleration. In capturing high dynamic motion of human limbs, the attitude angles are mainly estimated by integral of gyroscope output rather than gravity acceleration via accelerometer due to highly dynamic interference, and thus suffers from drift problem. The model-based data fusion method treats attitude estimation as a separation problem of gravity acceleration from motion acceleration according to their discrepant dynamic models. However, it is hard to establish a robust acceleration model that is applicable for diverse scenarios because the model is actually motion type dependent. Various improved data fusion methods by incorporating the model-based method with the threshold approach have been reported in recent years. For example, adaptive filter methods^38,39^ adopt adaptively regulating covariance matrix of noise to regulate the weight of the accelerometer in a model-based data fusion algorithm^26^, which are able to deal with transient or short-term dynamic motion capture. However, similar to the threshold-based method, attitude estimation using adaptive filter mostly or even only relies on integral of gyroscope in highly dynamic motion. Therefore, the adaptive filter methods using low-cost micro inertial sensors suffer from serious drift problem in capturing long-term dynamic motion. Although great efforts have been made in data fusion algorithm for inertial sensors^26,34–39^, inherent problems of drift and instability in long-term monitoring of highly dynamic limb motions still exist^40^, for example limb posture capture in running. To avoid dynamic interference, the inertial sensors are usually mounted on the trunk of human or robots^25,41^ instead of limbs. Current reports of soft exosuit show the utilization of inertial sensors in leg movement monitoring, but only for gait recognition^25^. Elevation angles of leg are estimated only in static or quasi-static cases (for example stand up and sit down)^42^. In addition, complexity and cost of wearable device is another sensitive issue to be considered. Reducing wearing nodes and lightening weights are important for next generation of wearable system. To solve the problem, a wearable motion capture device using dual-axis velocity sensor integrating with inertial sensors has been proposed to detect two-dimensional motion of limb in our previous articles^40,43^. A micro flow sensor was used to detect a motion-induced surface flow, by which two-dimensional motion velocity was determined.

**Fig. 1:**
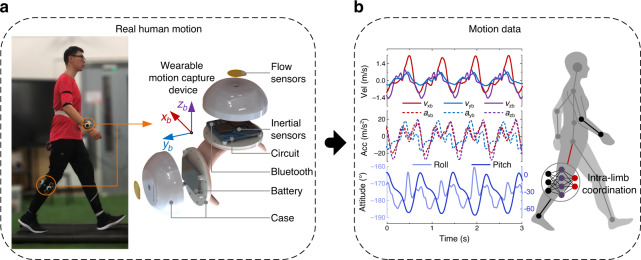
Design of the motion capture scheme. **a** Our wearable device is worn on human body segments of interest for motion capture by incorporating tri-axis flow sensors with tri-axis inertial sensors. **b** Motion data including three-dimensional motion velocity, motion acceleration, and attitude angles can be measured by our device. The motion velocity and motion acceleration are measured via integral-free approach by using micro flow sensor which avoids accumulative errors. The attitude angles are then accurately determined by incorporating the motion velocity and acceleration detected by the flow sensor with inertial quantities detected by the inertial sensors. Therefore, drift and instability problems are overcome. A neural network model is established to characterize the natural intra-limb coordination for human lower limb and used to determine the thigh motion from the shank motion in human walking and running.

In this work, we propose using combined micro flow sensors to detect tri-axis motion velocity and develop a wearable motion capture device by incorporating tri-axis flow sensors with tri-axis inertial sensors to implement accurate and robust three-dimensional motion measurement for human limbs with the simplest setup, as shown in Fig. 1a. The motion velocity and acceleration are measured via integral-free approach by using micro flow sensor, which avoids accumulative errors and thus overcomes drift and instability problems. We also design a data fusion algorithm to determine attitude angles by incorporating the motion velocity detected by the flow sensor with inertial quantities detected by the inertial sensors. Therefore, the developed wearable device is competent to accurately measure three-dimensional velocity, acceleration, and attitude angles of limbs in dynamic motions, as shown in Fig. 1b. In addition, we study the intra-limb coordination relationship between shank and thigh in human walking and running, and find the natural coordination model for human lower limb. We establish a neural network model to characterize the intra-limb coordination for human lower limb, and use it to determine the thigh motion from the shank motion in human walking and running, as shown in Fig. 1b. Thereby, people only need to wear single device on shank, while capably detect motions of both shank and thigh in real time. This configuration greatly simplifies the motion capture system, and reduces the cost and alignment complexity of wearable devices. To evaluate the performance of the device, we conduct a variety of limb motion captures for subjects who are doing boxing and kicking activities like Chinese Kungfu, and long-time walking and running. The experimental results validate effectiveness and superiority of accuracy and long-term stability of the device. In a word, we achieve accurate and robust limb motion capture in highly dynamic activities of human body using a simple wearable device.

## Results

### Wearable motion capture device

Inspired by the lateral line system of fish and amphibian animals for flow and motion sensing^44^, we propose a micro velocity sensor that measures motion velocity by detecting the motion-induced surface flow vectors using micro flow sensors. Two-dimensional (2-D) velocity measurement using the flow sensor has been proved in our previous works^40,43,45–47^. Here, we use two orthogonally-placed micro flow sensors to constitute a tri-axis velocity sensor, which further integrates with tri-axis accelerometer and tri-axis gyroscope to construct a wearable motion capture device (shown in Fig. 1a, named wearable device). More detailed design of the wearable device is described in “Methods” section. We propose an integral-free approach to determine three-dimensional motion velocity, acceleration and attitude angle of human limb to overcome cumulative errors. The tri-axis motion velocity **v**_b_ is measured by the velocity sensor based on flow detection. The tri-axis motion acceleration **a**_b_ is estimated from Eq. (1) by linear algebraic operation of **v**_b_ and tri-axis angular rate **ω**_b_ measured by the gyroscope (shown in Fig. 1b). Here, subscript *b* refers to the body reference frame of the wearable device.

1 $${\mathbf{a}}_{\mathrm{b}} = {\mathbf{\omega }}_{\mathrm{b}} \times {\mathbf{v}}_{\mathrm{b}} + {\dot{\mathbf{v}}}_{\mathrm{b}}$$

**Fig. 2:**
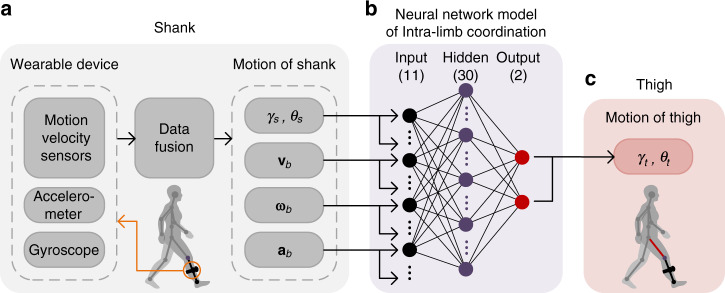
The principle of lower limb motion capture by single wearable device. **a** The motion of shank is directly measured by the device worn on the shank. **b** The neural network model of intra-limb coordination between shank and thigh has 30 hidden neurons and use motion information of shank as the inputs of the network (including shank attitude angles *γ*_s_, *θ*_s_, motion velocity **v**_b_, and the corresponding derivatives angular rate **ω**_b_ and motion acceleration **a**_b_) and use attitude angles of thigh (*γ*_t_, *θ*_t_) as the outputs of the network. **c** The thigh motion is determined from the shank motion measured by the wearable device incorporating with the intra-limb coordination model.

The attitude angles of limb are figured out by using a tailor-designed data fusion method incorporating the motion velocity detected by the flow sensor with the inertial quantities detected by the accelerometer and gyroscope (shown in Fig. 1b and Fig. 2a). In virtue of no integral operation in the calculation, none of accumulative errors is involved in motion velocity, acceleration and attitude estimations. In other words, the wearable capture device has competence to detect accurate motion velocity, acceleration and attitude angles for human limbs in dynamic motion (shown in Fig. 1).

### Intra-limb coordination model of human lower limb

Lower limb motion capture accounts for a high level importance in diagnose and rehabilitation of motor dysfunction, athlete training, and human-robot coordination in assistive robotic devices for locomotion^48–51^. In conventional methods^24,46^, at least two devices need to be worn separately on thigh and shank to measure the motion of lower limb. Alignment of the devices on two segments of lower limb is troublesome. As a matter of fact, a natural intra-limb coordination exists between shank and thigh motions in human walking and running, which has been evidenced by neurological and biomechanical researches due to nerve center control and anatomical/biomechanical constraint^52^. For example, a planar covariation law has been observed for lower limb that describes the coordination patterns among the elevation angles of the lower limb segments during locomotion^52–55^. In our study, we validate a natural intra-limb coordination relationship generally exists between thigh and shank in human walking and running. The intra-limb coordination helps to solve the inverse kinematic problem, as shown in Fig. 2.

To model this intra-limb coordination, we learn human neural system and recognize that neural network is an appropriate structure to model this dynamic behavior. The neural network has been applied for complex behavior modeling of actuators and robust control in humanoid robots^41,56^. Here, we use a three-layer back propagation (BP) neural network to model intra-limb coordination between shank and thigh motions in walking and running, as shown in Fig. 2b. Attitude angle (elevation angle), motion velocity, angular rate and motion acceleration of the shank are designated as inputs of the network. And the attitude angles of the thigh are as outputs of the network. The number of hidden neurons is determined to be 30 by an optimization described in “Methods” section. The neural network is trained and validated by using abundant motion datasets of shank and thigh in human walking and running captured by using an optical system (VICON VERO, Vicon Motion Systems Ltd). The established coordination model is then used to determine the thigh motion from the shank motion in the real-time monitoring of human lower limb motion. In other words, the motion capture of human lower limb (both shank and thigh) is achieved by using single device worn on shank incorporating with the neural network model of intra-limb coordination (shown in Fig. 2). The method simplifies the motion capture system, and reduces cost and complexity of wearable devices.

### Data fusion approach for measurements of the wearable device

As reported in our previous work^43^, 2-D motion velocity can be measured using a micro flow sensor. In this work, two micro flow sensors (shown in Fig. 1a) are orthogonally placed to achieve tri-axis motion velocity measurement which is derived according to Eq. (2), where $$\left[ {\begin{array}{*{20}{c}} {{\it{v}}_{{\mathrm{1bx}}}} \\ {{\it{v}}_{{\mathrm{1by}}}} \end{array}} \right]$$ and $$\left[ {\begin{array}{*{20}{c}} {{\it{v}}_{{\mathrm{2bx}}}} \\ {{\it{v}}_{{\mathrm{2bz}}}} \end{array}} \right]$$are the two-axis motion velocity measured by the two micro flow sensors, respectively.

2 $${\mathbf{v}}_{\mathrm{b}}{\mathrm{ = }}\left[ {\begin{array}{*{20}{c}} {\left( {{\it{v}}_{{\mathrm{1bx}}}{\it{ + v}}_{{\mathrm{2bx}}}} \right)/2} \\ {{\it{v}}_{{\mathrm{1by}}}} \\ {{\it{v}}_{{\mathrm{2bz}}}} \end{array}} \right]$$

The relationship between tri-axis motion velocity **v**_b_ and tri-axis motion acceleration **a**_b_ is complex due to their different decomposition criteria in body reference frame *x*_b_*y*_b_*z*_b_. The following derivation from the geographic reference frame *x*_n_*y*_n_*z*_n_ is formulated by

3 $${\mathbf{a}}_{\mathrm{n}} = {\dot{\mathbf{v}}}_{\mathrm{n}}$$

Assuming the orientation matrix from *x*_n_*y*_n_*z*_n_ to *x*_b_*y*_b_*z*_b_ is ${\mathbf{T}}_{\mathrm{n}}^{\mathrm{b}}$, the orientation matrix from *x*_b_*y*_b_*z*_b_ to *x*_n_*y*_n_*z*_n_ is therefore ${\mathbf{T}}_{\mathrm{b}}^{\mathrm{n}} = {\mathbf{T}}_{\mathrm{n}}^{{\mathrm{b - 1}}}$. The motion velocity and acceleration in *x*_n_*y*_n_*z*_n_ and *x*_b_*y*_b_*z*_b_ satisfy Eqs. (4) and (5).

4 $${\mathbf{v}}_{\mathrm{n}} = {\mathbf{T}}_{\mathrm{b}}^{\mathrm{n}}{\mathbf{v}}_{\mathrm{b}}$$

5 $${\mathbf{a}}_{\mathrm{b}} = {\mathbf{T}}_{\mathrm{n}}^{\mathrm{b}}{\mathbf{a}}_{\mathrm{n}}$$

Substitute Eq. (3) into Eq. (5), there is

6 $${\mathbf{a}}_{\mathrm{b}} = {\mathbf{T}}_{\mathrm{n}}^{\mathrm{b}}{\dot{\mathbf{v}}}_{\mathrm{n}}$$

Equation (7) is derived by substituting Eq. (4) into Eq. (6).

7 $${\mathbf{a}}_{\mathrm{b}} = {\mathbf{T}}_{\mathrm{n}}^{\mathrm{b}}{\dot{\mathbf{T}}}_{\mathrm{b}}^{\mathrm{n}}{\mathbf{v}}_{\mathrm{b}} + {\dot{\mathbf{v}}}_{\mathrm{b}}$$

According to the character of orientation matrix, it is validated that

8 $${\dot{\mathbf{T}}}_{\mathrm{b}}^{\mathrm{n}} = {\mathbf{T}}_{\mathrm{b}}^{\mathrm{n}}{\mathrm{[}}{\mathbf{\omega }}_{\mathrm{b}} \times {\mathrm{]}}$$

where $$\left[ {{\mathbf{\omega }}_{\mathrm{b}} \times } \right] = \left[ {\begin{array}{*{20}{c}} {\mathrm{0}} & {{\mathrm{ - }}\omega _{{\mathrm{bz}}}} & {\omega _{{\mathrm{by}}}} \\ {\omega _{{\mathrm{bz}}}} & {\mathrm{0}} & {{\mathrm{ - }}\omega _{{\mathrm{bx}}}} \\ {{\mathrm{ - }}\omega _{{\mathrm{by}}}} & {\omega _{{\mathrm{bx}}}} & {\mathrm{0}} \end{array}} \right]$$ is the skew-symmetric matrix of angular rate in body reference frame $${\mathbf{\omega }}_{\mathrm{b}} = \left[ {\begin{array}{*{20}{c}} {\omega _{{\mathrm{bx}}}} & {\omega _{{\mathrm{by}}}} & {\omega _{{\mathrm{bz}}}} \end{array}} \right]^{\mathrm{T}}$$, which can be measured by the tri-axis gyroscope.

The relationship between **v**_b_ and **a**_b_ is therefore achieved by substituting Eq. (8) into Eq. (7), where the operator × is cross product of vector.

9 $${\mathbf{a}}_{\mathrm{b}} = \left[ {{\mathbf{\omega }}_{\mathrm{b}} \times } \right]{\mathbf{v}}_{\mathrm{b}} + {\dot{\mathbf{v}}}_{\mathrm{b}} = {\mathbf{\omega }}_{\mathrm{b}} \times {\mathbf{v}}_{\mathrm{b}} + {\dot{\mathbf{v}}}_{\mathrm{b}}$$

According to Eq. (9), the tri-axis motion acceleration of our device can be determined without any accumulative error by linear algebra operation of tri-axis velocity measured by the micro flow sensor and tri-axis angular rate measured by gyroscope.

In theory, the accelerometer output vector **f**_b_ is the total acceleration including the gravity acceleration **g**_b_ and the motion acceleration **a**_b_.

10 $${\mathbf{g}}_{\mathrm{b}} = {\mathbf{f}}_{\mathrm{b}} + {\mathbf{a}}_{\mathrm{b}}$$

To determine attitude angles with robust performance of anti-interference, we propose a tailor-designed filter algorithm considering natural dynamics and inherent correlation between motion velocity and acceleration to implement data fusion of the motion velocity detected by the flow sensor and inertial quantities detected by the accelerometer and gyroscope. The data fusion of velocity sensor and inertial sensors aims to suppress the errors of the sensors (e.g., noise of velocity sensor, shock and vibration interference in accelerometer, bias and noise of gyroscope). Motion velocity and motion acceleration are defined as the first part of state variables

11 $${\mathbf{X}}_{\mathrm{1}} = \left[ {\begin{array}{*{20}{c}} {{\mathbf{v}}_{\mathrm{b}}} \\ {{\mathbf{a}}_{\mathrm{b}}} \end{array}} \right]$$

Here, a stochastic modeling approach using Gauss–Markov (GM) model is adopted in this work to model the dynamic behavior of the motion acceleration, as shown in Eq. (12)^26^, where, **w**_1_ is white Gaussian noise having zero mean and standard deviation *σ*_w1_ for each component, *η* is constant.

12 $${\dot{\mathbf{a}}}_{\mathrm{b}} = \eta {\mathbf{a}}_{\mathrm{b}} + {\mathbf{w}}_1$$

According to Eqs. (9), (11), and (12), one continuous state equation model is established as shown in Eq. (13) where **w**_g_ is the process error caused by gyroscope. And the corresponding discrete-time model is shown in Eq. (14), where superscript *k* is the *k*th time sample, *T*_s_ is the sampling period, ${\mathbf{W}}_{\mathrm{1}}^k$is the corresponding process noise whose covariance matrix ${\mathbf{Q}}_{\mathrm{1}}^k$ is derived from Eq. (15), *σ*_g_ is the standard deviation of gyroscope measurement, $\left[ {{\mathbf{v}}_{\mathrm{b}} \times } \right]$ is the skew-symmetric matrix of **v**_b_.

13 $${\dot{\mathbf{X}}}_{\mathrm{1}} = \left[ {\begin{array}{*{20}{c}} { - [{\mathbf{\omega }}_{\mathrm{b}} \times ]} & {{\mathbf{I}}_{\mathrm{3}}} \\ {{\mathbf{0}}_3} & {\eta {\mathbf{I}}_3} \end{array}} \right]{\mathbf{X}}_1 + \left[ {\begin{array}{*{20}{c}} {{\mathbf{w}}_{\mathrm{g}}} \\ {{\mathbf{w}}_1} \end{array}} \right] = {\mathbf{A}}_1{\mathbf{X}}_1 + \left[ {\begin{array}{*{20}{c}} {{\mathbf{w}}_{\mathrm{g}}} \\ {{\mathbf{w}}_1} \end{array}} \right]$$

14 $${\mathbf{X}}_1^{k + 1} = {\mathrm{e}}^{{\mathbf{A}}_1T_s}{\mathbf{X}}_1^k + {\mathbf{W}}_1^k$$

15 $${\mathbf{Q}}_1^k \approx \left[ {\begin{array}{*{20}{c}} {\sigma _{\mathrm{g}}^2T_{\mathrm{s}}^{\mathrm{2}}\left[ {{\mathbf{v}}_{\mathrm{b}} \times } \right]^k\left( {\left[ {{\mathbf{v}}_{\mathrm{b}} \times } \right]^k} \right)^{\mathrm{T}}} & {{\mathbf{0}}_3} \\ {{\mathbf{0}}_3} & {\sigma _{{\mathrm{w1}}}^{\mathrm{2}}T_{\mathrm{s}}^{\mathrm{2}}{\mathbf{I}}_3} \end{array}} \right]$$

Gravity acceleration **g**_b_ needs to be estimated for decoupling with motion acceleration **a**_b_. Therefore, **g**_b_ is defined as the second part of the state variables **X**_2_ = **g**_b_ whose evolution is described by the following differential equation^36^

16 $${\dot{\mathbf{g}}}_{\mathrm{b}} = - \left[ {{\mathbf{\omega }}_{\mathrm{b}} \times } \right]{\mathbf{g}}_{\mathrm{b}}$$

The discrete-time state equation model of Eq. (16) is established as shown in Eq. (17) under the assumption that the angular rate is constant in the sampling period, where ${\mathbf{W}}_2^k$ is the corresponding process noise and its covariance matrix ${\mathbf{Q}}_2^k$ is derived from Eq. (18), $\left[ {{\mathbf{g}}_{\mathrm{b}} \times } \right]$ is the skew-symmetric matrix of **g**_b_.

17 $${\mathbf{X}}_2^{k + 1} = {\mathrm{e}}^{( - [{\mathbf{\omega }}_{\mathrm{b}} \times ]^kT_{\mathrm{s}})}{\mathbf{X}}_2^k + {\mathbf{W}}_2^k$$

18 $${\mathbf{Q}}_2^k \approx \sigma _{\mathrm{g}}^2T_{\mathrm{s}}^{\mathrm{2}}\left[ {{\mathbf{g}}_{\mathrm{b}} \times } \right]^k\left( {\left[ {{\mathbf{g}}_{\mathrm{b}} \times } \right]^k} \right)^{\mathrm{T}}$$

The measurement variables are defined as the tri-axis motion velocity (**v**_bm_) and total acceleration (**f**_bm_) measured by the micro velocity sensors and the accelerometer, respectively, formulated in Eq. (19). And the measurement equation is therefore established as shown in Eq. (20). ${\mathbf{\upsilon }}_1$ and ${\mathbf{\upsilon }}_2$ are measurement noises (assumed as white Gaussian noises) of the micro velocity sensors and the accelerometer, respectively, having zero mean and standard deviation $\sigma _{{\upupsilon 1}}$ and $\sigma _{{\upupsilon 2}}$, respectively.

19 $${\mathbf{Y}} = \left[ {\begin{array}{*{20}{c}} {{\mathbf{v}}_{{\mathrm{bm}}}} \\ {{\mathbf{f}}_{{\mathrm{bm}}}} \end{array}} \right]$$

20 $${\mathbf{Y}} = \left[ {\begin{array}{*{20}{c}} {\begin{array}{*{20}{c}} {{\mathbf{I}}_3} & {{\mathbf{0}}_3} & {{\mathbf{0}}_3} \end{array}} \\ {\begin{array}{*{20}{c}} {{\mathbf{0}}_3} & { - {\mathbf{I}}_3} & {{\mathbf{I}}_3} \end{array}} \end{array}} \right]\left[ {\begin{array}{*{20}{c}} {{\mathbf{X}}_1} \\ {{\mathbf{X}}_2} \end{array}} \right] + \left[ {\begin{array}{*{20}{c}} {{\mathbf{\upsilon }}_1} \\ {{\mathbf{\upsilon }}_2} \end{array}} \right] = {\mathbf{CX}} + {\mathbf{\upsilon }}$$

Combining Eqs. (14), (17), and (20), the discrete-time state-space models of Kalman filter are obtained as follows

21 $${\mathbf{X}}^{k + 1} = {\mathbf{{\Phi} }}^k{\mathbf{X}}^k + {\mathbf{W}}^k$$

22 $${\mathbf{Y}}^{k + 1} = {\mathbf{C}}^k{\mathbf{X}}^k + {\mathbf{\upsilon }}^k$$

where $${\mathbf{{\Phi} }}^k = \left[ {\begin{array}{*{20}{c}} {{\mathrm{e}}^{{\mathbf{A}}_1T_{\mathrm{s}}}} & {{\mathbf{0}}_3} \\ {{\mathbf{0}}_3} & {{\mathrm{e}}^{( - [{\mathbf{\omega }}_{\mathrm{b}} \times ]^kT_{\mathrm{s}})}} \end{array}} \right]$$, $${\mathbf{W}}^k = \left[ {\begin{array}{*{20}{c}} {{\mathbf{W}}_1^k} \\ {{\mathbf{W}}_2^k} \end{array}} \right]$$. The covariance matrix ${\mathbf{Q}}^k$ of the process noise ${\mathbf{W}}^k$ is formulated as Eq. (23)

23 $${\mathbf{Q}}^k = \left[ {\begin{array}{*{20}{c}} {{\mathbf{Q}}_1^k} & {{\mathbf{0}}_{6 \times 3}} \\ {{\mathbf{0}}_{3 \times 6}} & {{\mathbf{Q}}_2^k} \end{array}} \right]$$

The covariance matrix ${\mathbf{R}}^k$ of the measurement noise ${\mathbf{\upsilon }}^k$ is calculated according to Eq. (24) with zero non-diagonal elements, under the assumption that both the process noises and measurement noises are uncorrelated with each other.

24 $${\mathbf{R}}^k = {\mathbf{\upsilon }}^k({\mathbf{\upsilon }}^k)^{\mathrm{T}}$$

**Fig. 3:**
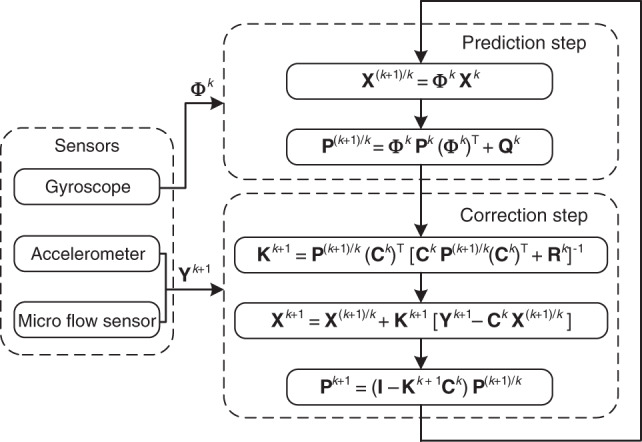
The implementation process of the data fusion algorithm. **P**^*(k*+1*)/k*^ is state vector prediction error, **P**^*k*^ is the error of the filter output in the *k*th iteration, **K**^*k*+1^ is the filter gain.

The implementation process of the data fusion algorithm is shown in Fig. 3, where ${\mathbf{P}}^{(k + 1)k}$ is the state vector prediction error, **P**^*k*^ is the error of the filter output in the *k*th iteration, ${\mathbf{K}}^{k + 1}$ is the filter gain.

The roll angle *γ* and pitch angle *θ* are calculated by Eq. (25)^57^, using the gravity acceleration components in the body frame $${\mathbf{g}}_{\mathrm{b}} = \left[ {\begin{array}{*{20}{c}} {g_{{\mathrm{bx}}}} & {g_{{\mathrm{by}}}} & {g_{{\mathrm{bz}}}} \end{array}} \right]^{\mathrm{T}}$$ determined by the proposed data fusion algorithm.

25 $$\left\{ {\begin{array}{*{20}{c}} {\gamma = \tan ^{ - 1}\left( {\frac{{g_{{\mathrm{by}}}}}{{g_{{\mathrm{bz}}}}}} \right)} \\ {\theta = - \tan ^{ - 1}\left( {\frac{{g_{{\mathrm{bx}}}}}{{g_{{\mathrm{bz}}}}}\cos \gamma } \right)} \end{array}} \right.$$

Therefore, three-dimensional velocity **v**_b_ can be measured, and three-dimensional acceleration **a**_b_ and attitude angles (*γ* and *θ*) can be accurately determined by using the developed wearable device and the proposed data fusion approach.

### Dynamic motion capture in strenuous exercises

High dynamic motion of human limbs happens not only when performing daily activities such as walking and running but also when doing strenuous exercises, for example boxing and kicking. To validate the motion capture performance of the wearable device in monitoring highly dynamic motions, experiments on motion capture of human upper and lower limbs are carried out in vigorous activities of Kongfu. A subject wears a device on his wrist and shank, respectively. He conducts boxing continuously for about 1 min and then plays kicking for another 1 min. The motion capture results of our device are compared with the results of optical VICON system detected synchronously. A conventional inertial method using typical model-based Kalman filter for data fusion of accelerometer and gyroscope^26^ is also adopted to make a comparison with our device. This inertial method adopts a first-order GM model similar to Eq. (12) to represent the dynamic behavior of the motion acceleration. And data fusion of accelerometer and gyroscope is implemented by their complementary characters based on Kalman filter to estimate the motion acceleration and the gravity acceleration.

**Fig. 4:**
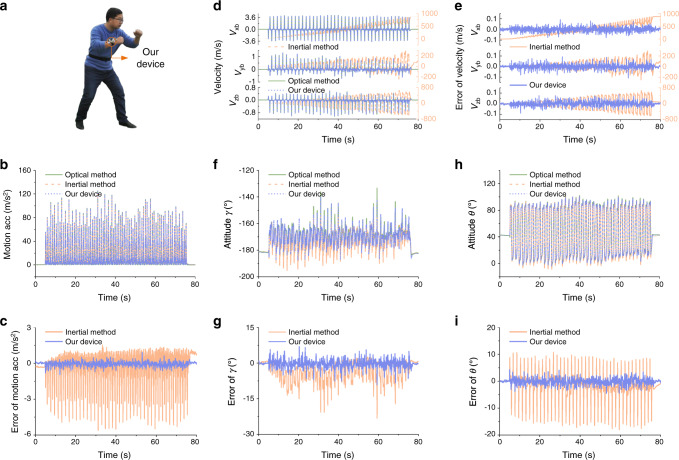
Boxing motion capture results of forearm wearing a device. **a** The subject wears a device on his wrist and conducts boxing activity. **b**–**c** The magnitude of motion acceleration measured by our device and inertial method, respectively (**b**), and the corresponding errors (**c**). High dynamic motion with motion acceleration more than 120 m/s^2^ is observed. **d**–**e** The tri-axis motion velocity measured by our device and inertial method, respectively (**d**), and the corresponding errors (**e**). **f**–**i** The attitude estimation results of roll angle *γ* (**f**) and pitch angle *θ* (**h**), respectively, and the corresponding attitude errors by our device and inertial method respectively (**g** and **i**). Motion capture results of optical VICON system are used as reference values.

**Fig. 5:**
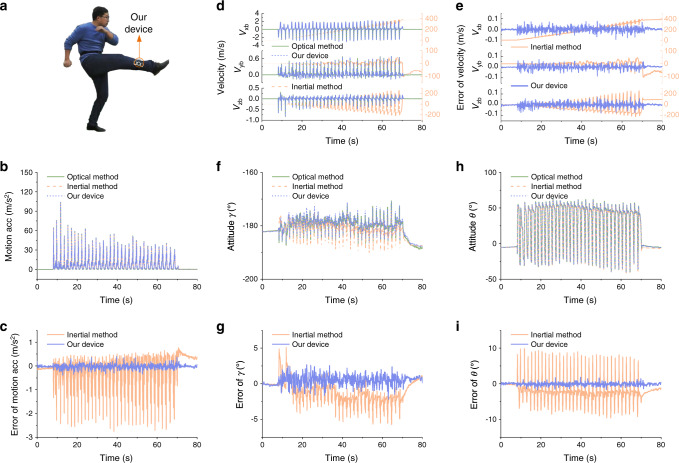
Kicking motion capture results of shank wearing a device. **a** The subject wears a device on his shank and conducts kicking activity. **b**–**c** The magnitude of motion acceleration measured by our device and inertial method, respectively (**b**), and the corresponding errors (**c**). High dynamic motion with motion acceleration more than 100 m/s^2^ is observed. **d**–**e** The tri-axis motion velocity measured by our device and inertial method, respectively (**d**), and the corresponding errors (**e**). **f**–**i** The attitude estimation results of roll angle *γ* (**f**) and pitch angle *θ* (**h**), respectively, and the corresponding attitude errors by our device and inertial method, respectively (**g** and **i**). Motion capture results of optical VICON system are used as reference values.

The experimental results of the forearm and shank motion capture in boxing and kicking motions are shown in Fig. 4 and Fig. 5, respectively. The motion capture performance is summarized in Supplementary Table 1. High dynamics of boxing and kicking are observed, the motion acceleration in boxing and kicking exceeds 120 m/s^2^ and 100 m/s^2^, respectively, as shown in Fig. 4b and Fig. 5b. The result of the magnitude of motion acceleration measured using our device is consistent with that of the optical VICON, whose error is obviously less than that using the conventional inertial method as shown in Fig. 4c and Fig. 5c. The measurement results of tri-axis motion velocity are shown in Fig. 4d and Fig. 5d, where the measurement error of velocity using our device is only less than 0.11 m/s, and the results of conventional inertial method^30^ exhibit unbounded drift errors shown in Fig. 4e and Fig. 5e. Results also indicate that the measurement errors of attitude angles determined by our device are greatly less than that of the inertial method. Specifically, the root-mean-square error (RMSE) of attitude angles by our device is less than 1.70°, which halves the error of the inertial method. And the drift error of attitude angles using our device is negligible as the mean error (ME) is less than 0.47°. In contrast, the drift error of attitude angles by the conventional inertial method is larger, the ME reaches 3.19° and the RMSE reaches 4.18° as shown in Fig. 4g, i, Fig. 5g, i and Supplementary Table 1.

### Motion capture in long-time running

**Fig. 6:**
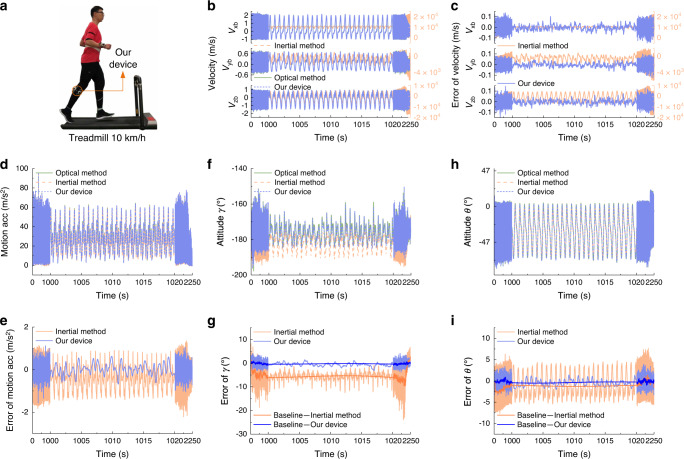
Long-time motion capture and velocity measurement results. **a** The subject wearing a device on his shank keeps running on a treadmill at the speed of 10 km/h for about 30 min and then gradually stop for another 7 min. **b**–**c** The tri-axis motion velocity measured by our device and inertial method, respectively (**b**), and the corresponding errors (**c**). **d**–**e** The magnitude of motion acceleration measured by our device and inertial method, respectively (**d**), and the corresponding errors (**e**). High dynamic motion with motion acceleration more than 70 m/s^2^ is observed. **f**–**i** The attitude estimation results of roll angle *γ* (**f**) and pitch angle *θ* (**h**), respectively, and the corresponding attitude errors by our device and inertial method, respectively (**g** and **i**). Motion capture results of optical VICON system are used as reference values.

A long-time motion capture experiment of human running on a treadmill is further conducted to evaluate the long-term stability and accuracy of our device. An experienced athlete wearing our device on his shank keeps running on a treadmill at a speed of 10 km/h for about 30 min (Fig. 6a) and then gradually slows down for about 7 min until stop. The optical VICON system is used synchronously to provide reference of motion. As a comparison, the conventional inertial method is also used to detect attitude angles of shank.

The experimental results of velocity, acceleration and attitude angle measurements are shown in Fig. 6. The velocity results shown in Fig. 6b, c indicate that the measurement errors of velocity using our device keep less than 0.16 m/s in the long-time motion, whereas the velocity errors using the inertial method drift over time and reach to myriametre per second. The results of attitude angles shown in Fig. 6g, i indicate that the attitude angles determined by our device have negligible drift error. The RMSE is less than 0.84° and the maximum attitude estimation error of our device is less than 4.12°, while that of inertial method reaches 24.01°. The inertial method exhibits obvious drift in estimation of attitude angles. The baseline drift of attitude estimation is evaluated using Butterworth low-pass filter with cut-off frequency of 0.05 Hz. At the end of the running for 30 min, the roll angle and pitch angle of the shank determined by inertial method exhibit the maximum baseline drift error of −9.7°. Removing the baseline drift, the RMSE of the residual error is 1.73° and 2.83° respectively for roll and pitch angles using the inertial method.

### Lower limb motion capture by wearing single device on shank

Four subjects with different ages including one patient suffering from mild meniscus injury participate in the experiments. First, an intra-limb coordination model of shank and thigh motions in walking and running is established for each subject. The neural network model training is described in “Methods” section. And then, we use single device worn on subject shank to capture motions of the whole lower limb. The thigh motion is determined from the shank motion detected by our device according to the intra-limb coordination model. In rehabilitation robot application, especially wearable assistive soft-exosuit, the elevation angles of lower limb (i.e., attitude angles) and joint angle of knee (i.e., knee angle) in the sagittal plane and the coronal plane are requisite for automatic control of assistive locomotion. The elevation angles and joint angle of the lower limb are estimated in real time from the attitude angles of the thigh and shank. We conduct validation experiments to monitor elevation angles and joint angle of lower limb in human walking and running. Three repeated validation experiments are carried out for each subject who repeatedly walks and runs on a treadmill with a velocity increasing from 0 to 10 km/h at an interval of 1 km/h. Subject 2 to 4 carry out three repeated experiments continuously for about 26 min without any rest, while Subject 1 takes about 5-mins rest between repeated experiments.

**Fig. 7:**
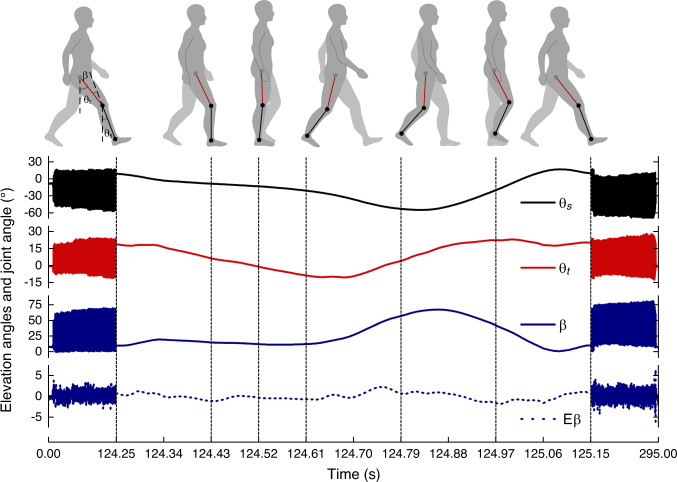
Results of lower limb motion capture in the sagittal plane by using single device worn on the shank and determining the thigh motion from the shank motion by the trained neural network model of intra-limb coordination. The pitch angles of thigh *θ*_t_ and shank *θ*_s_ are used to represent the corresponding elevation angles in the sagittal plane, respectively. The joint angle of knee *β* in the sagittal plane is derived by subtracting *θ*_s_ from *θ*_t_. The error of *β* (E*β*) represents the estimation performance of both *θ*_t_ and *β*, including the measurement error of the wearable device and the model error of the neural network.

An experiment result for Subject 1 is shown in Fig. 7. All experiment results for four subjects are shown in Supplementary Figs. 1 to 4. The pitch angles of thigh *θ*_t_ and shank *θ*_s_ represent the corresponding elevation angles of segments of lower limb in the sagittal plane, respectively and the joint angle of knee *β* is calculated by subtracting *θ*_s_ from *θ*_t_. The deviation error of the joint angle measured by our device from that of the optical VICON system is denoted as the knee error E*β*, which is used to evaluate the measurement accuracy of the limb motion capture. Error results of four subjects are summarized in Supplementary Table 2. The ME of knee angle is less than 0.66° and the RMSE is less than 1.20° for all subjects. The results indicate that different people, even the patient with knee injury (Subject 4), have their own intra-limb coordination relationship between thigh and shank during walking and running. The results also verify that the neural network model enables to represent the intra-limb coordination relationship that can be used to determine the thigh motion from the shank motion in human walking and running.

The influence of physical condition (e.g., fatigue) on intra-limb coordination model of each subject is further analyzed. The maximum deflection of shank |*θ*_s_|^peaks^ of different subjects in each repeated experiment of lower limb motion capture is estimated and shown in Supplementary Fig. 5, which is evaluated by averaging peak values of |*θ*_s_| during the last 25 s running on a treadmill at velocity of 10 km/h. Maximum deflection of shank reveals the ability of lifting heel and can be used as an indicator of fatigue^58^. For Subject 1, his maximum deflection of shank in three repeated experiments keeps consistent due to having rest between repeated experiments. For Subject 2 to 4, their maximum deflections of shanks show similar declining trend with time, which indicates the fatigue increases gradually in continuously running for nearly half an hour. Despite variation existing in physical condition (e.g., fatigue) of subjects, the proposed intra-limb coordination model for each subject keeps working well as shown in Supplementary Table 2. The motion capture results of four subjects validate the effectiveness of the intra-limb coordination model. In addition, human gait pattern can be also explicitly recognized according to the elevation angles of lower limb as shown in Fig. 7. Using single device to capture motion and gait pattern of lower limb significantly simplifies the monitoring system, reduces cost and lightens wearable devices.

Moreover, the maximum knee angle of the patient (Subject 4) suffering from motor function injury is compared with the other three healthy subjects (Subject 1 to 3), which is shown in Supplementary Fig. 6. It can be seen that the maximum knee angle of the patient with meniscus injury is smaller than that of the healthy people in both walking and running. People with knee injury suffer from weakened knee flexion ability and thus exhibit smaller maximum knee angles than healthy people in walking and running due to pain or pathological knee constraints^59^. Therefore, maximum knee angle can be used as an indicator of knee flexion ability that has potential in diagnosis and assessment of motor dysfunction or injury.

## Discussion

Above experimental results demonstrate that the proposed approach using the wearable device are accurate, reliable and robust for dynamic motion capture of human limbs even in strenuous exercises like boxing and kicking. Three-dimensional motion velocity of limb is measured accurately in real time, which facilitates to evaluate fine motor function of limb or perform limb control for robots and prosthetics. Conventional inertial method using accelerometer and gyroscope induces large errors in velocity and attitude estimations when capturing highly dynamic motion. The reason is that the accelerometer output is dramatically fluctuated in highly dynamic motions, the attitude estimation mainly relies on the integral calculation of the angular rate measured by the gyroscope that results in accumulative errors. Therefore, the inertial sensors are usually mounted on the trunks instead of limbs to avoid dynamic interferences^25,41^ or only applicable to gait recognition^25^. In contrast, our device measures the motion velocity and motion acceleration by using the flow sensors. The velocity and attitude angles are estimated without the necessity of integral calculation, and thus achieve drift-free and robust measurements. An excellent stability and high accuracy are achieved by our device when capturing dynamic motion of human limbs in daily activities, such as walking, running, jumping and stepping.

The proposed intra-limb coordination model is an effective solver to seek the optimal solution of the inverse kinematic problem between shank and thigh by approximation. We establish a neural network model to characterize the intra-limb coordination of human lower limb. Attitude angles, angular rate, motion velocity and acceleration of distal segment (shank) are used as inputs of the network, and attitude angles of the proximal segment (thigh) are used as outputs. The motion capture experiments of four subjects who walk and run at different speeds validate the effectiveness and robustness of the intra-limb coordination model. The intra-limb coordination model helps to determine the thigh motion from the shank motion. Wearing single device on shank to capture motions of the whole lower limb is feasible, which simplifies the motion capture system, and reduces cost and complexity of wearable devices. Reduction of human metabolic penalty^25,60,61^ and device complexity using our proposed method is analyzed in Supplementary Fig. 7 and Supplementary Table 3. The intra-limb coordination based motion capture approach provides a simple and feasible way to solve the problem of dynamic motion capture for multiple limbs.

It is known that a neural network model is a data-driven method. To build an accurate model, a variety of samples need to be collected for training the network. In this work, the datasets are obtained by using an optical VICON system as a reference system. Due to limitations of the now available optical platform, e.g., occlusion problem, we have not conducted complex activities, for example the motions under complex terrain. The training of the neural network model is still time-consuming and computation-intensive, and needs to be improved. In addition, human intra-limb coordination model is probably individual dependent. There may exists difference between such as teenagers and elders, or healthy and disabled people. The coordination model may also exhibit similarity among different people when body parameters (e.g., height and weight) are taken into account. Comprehensive intra-limb coordination still needs to be investigated in future work.

Our proposed device provides a simple solution for robust monitoring of human limb motion in daily activities. It is simple to set up, and free of environmental restriction. Therefore, it has promising potential for the applications in fields of senior care, physical rehabilitation, daily life-logging, personal fitness and human-robot cooperation by incorporating with Internet of Things (IOT). Preliminary experiments demonstrate that characteristic indicators of knee flexion and shank deflection in human walking and running can be potentially used for health assessment or motion function evaluation. The challenges of making wearable motion capture devices for IOT based applications involve big data processing and transmission, low-power supply for sensor nodes, lowering weight/size and cost for personal uses, etc.

As mentioned above, we will conduct motion capture experiments on a variety of human activities to improve accuracy and robustness of the intra-limb coordination neural network model for practical applications. We will carry out researches on motion capture under complex terrain. Besides, the performance of the device for motion capture in the outdoor condition is going to be further investigated. To be mentioned that the indoor wind resistance of our device in measurements of motion acceleration and attitude angles has been validated in our previous works^43^. In future work, we will work on reducing the power consumption of the device by optimizing the sensor and circuit configuration. In addition, we will study human motor function assessment in daily life and are going to use it for diagnose and prevention of motor function injury. Research on applications in wearable exoskeleton for improving human-robot interaction will be also carried out in the future.

## Methods

### Design of the wearable device

In this work, we design a wearable motion capture device (shown in Supplementary Fig. 8a) able to accurately measure tri-axis motion velocity, tri-axis motion acceleration and attitude angles by incorporating a micro velocity sensor with inertial sensors. A homemade micro velocity sensor, comprising two micro flow sensors placed orthogonally, is used to measure tri-axis motion velocity by detecting the motion-induced surface flow vectors. The size and weight of micro velocity sensor is 10 × 10 × 0.05 mm^3^ and 0.024 g, respectively, while the total size and weight of the wearable device is 79 × 79 × 51 mm^3^ and 69.1 g, respectively. The accuracy (RMSE) of the micro velocity sensor is 0.16 m/s in motion velocity range of 0 to 3 m/s^43^. A commercial micro-inertial-measurement-unit (MIMU, LSM9DS1, STMicroelectronics) comprising tri-axis accelerometer and tri-axis gyroscope is selected to detect tri-axis acceleration and tri-axis angular velocity due to its low cost and small size. The measuring ranges of accelerometer and gyroscope are ±160 m/s^2^ and ±2000°/s, respectively to allow measurement of high dynamic motion. The accuracies (RMSE) of accelerometer and gyroscope are 0.021 m/s^2^ and 0.21°/s, respectively. A conditioning circuit based on constant temperature difference (CTD) feedback principle is designed for the homemade micro flow sensors. A low-power Bluetooth module (DA14580, Dialog Semiconductor) is used for wireless data transmission. A rechargeable lithium battery is used for power supply shown in Fig. 1. A watch-like case is designed for packaging the device, making it wearable. The schematic diagram of the wearable device is shown in Supplementary Fig. 8b, where the MIMU is connected with a micro control unit (MCU, STM32L476, STMicroelectronics) through serial peripheral interface (SPI). The outputs of the micro flow sensors and the MIMU are collected by the MCU at a sampling frequency of 1000 Hz, processed in the MCU and transmitted wirelessly to terminals (e.g., smart phone or PC) through Bluetooth at a frequency of 100 Hz. The micro flow sensor has power consumption of about 30 mW. The MIMU and the Bluetooth module have power consumption of 15.2 mW and 4 mW, respectively. The system circuit consisting of the sensors’ operation circuit, the MCU and the power management circuit has power consumption of about 400 mW. Therefore the total power consumption of the proposed wearable device is about 450 mW. The device can work continuously for more than 3.5 h utilizing a 600 mAh lithium battery. To reduce the power consumption of the device, the MCU intelligently manages the power supply. The MCU powers down the velocity sensors at quiescent state and wakes up them when detecting active motion by the accelerometer. Therefore, highly efficient power management of the device is achieved in real application.

The Forward-Left-Up (FLU) frame *x*_b_*y*_b_*z*_b_ (shown in Supplementary Fig. 8a) is defined as the body reference frame fixed on the wearable device, where *z*_b_ points to the upward direction of the device, *y*_b_ points to the left and *x*_b_ points to the forward. The geographic reference frame (*x*_n_*y*_n_*z*_n_) refers to North-West-Up (NWU), where *z*_n_ points to the opposite direction of gravity, *y*_n_ points to west, *x*_n_ points to north. Attitude angles of roll and pitch are denoted as *γ* and *θ*, which are used to represent the limb posture in the human motion capture experiment.

### Fabrication of micro flow sensor

The micro flow sensor (shown in Supplementary Fig. 9) is made by the following steps^62^: (i) Spin coating a 30 μm photoresist (KXN5735-LO, Rdmicro Co. Ltd.) on a polyimide substrate (AP8525R, DuPont Co. Ltd.). (ii) Obtaining the pattern by photolithography and development. (iii) Sputtering 30 nm thick chromium as an adhesion layer, and then sputtering 150 nm thick platinum as the thermo-sensitive layer. (iv) The patterned substrate is immersed in acetone for 2 h to dissolve the photoresist, and then washed with absolute ethanol and deionized water. (v) Deposit 4 μm thick parylene film on the patterned substrate as protective layer.

### Motion velocity calculation

As shown in Supplementary Figs. 9 and 10a, the micro flow sensor includes three central thermo-sensitive platinum ribbons (denoted as hot film R_hi_–R_hi_) and three circumambient thermo-sensitive platinum ribbons (denoted as cold film ${\mathrm{R}}_{{\mathrm{c}}1}$–${\mathrm{R}}_{{\mathrm{c}}3}$). The hot films are electrically heated and function as the flow sensors, the cold films act as the ambient temperature sensors and are used for temperature compensation for the hot films. A constant temperature difference (CTD) feedback circuit is adopted for each pair of hot film and cold film in the sensor (Supplementary Fig. 10b) to achieve temperature compensation^57,63,64^, where ${\mathrm{R}}_{{\mathrm{a}}1}$ and ${\mathrm{R}}_{{\mathrm{b}}1}$ are resistors for balancing the Wheatstone bridge for the first pair of hot and cold films ${\mathrm{R}}_{{\mathrm{h}}1}$ and ${\mathrm{R}}_{{\mathrm{c}}1}$, ${\mathrm{R}}_{{\mathrm{tb}}1}$ is for adjusting the Joule heating of hot film ${\mathrm{R}}_{{\mathrm{h}}1}$, and the feedback voltage *U*_1_ acts as the sensor output of ${\mathrm{R}}_{{\mathrm{h}}1}$. Similarly, *U*_2_ and *U*_3_ are the corresponding sensor outputs of ${\mathrm{R}}_{{\mathrm{h}}2}$ and ${\mathrm{R}}_{{\mathrm{h}}3}$. Thanks to the CTD circuit, the motion velocity detection is independent from variation of the environment temperature.

The working principle of the micro flow sensor for measuring planar motion velocity, illustrated in Supplementary Fig. 10a, is based on heat convection, where three isolated micro thermal elements (hot films) are electrically heated and measure motion-induced flow **v**_f_ blowing over the sensor. The planar motion velocity **v**_b_ is figured out according to Eq. (26)^43^, where *f*(*U*) is determined according to King’s law^65^ and *α* and *β* are constant parameters related to the geometric features and working conditions of the micro flow sensor^43^.

26 $$\left\{ {\begin{array}{*{20}{c}} {U = \frac{{U_1 + U_2/\alpha _1 + U_3/\alpha _2}}{3}} \\ {v_{\mathrm{b}} = - v_{\mathrm{f}} = - f\left( U \right)} \\ {\psi = \tan ^{ - 1}\left\{ {\frac{{\left( {\alpha _1\beta _2 - \alpha _2\beta _1} \right)U_1 + \left( {\alpha _2 + 2\beta _2} \right)U_2 - \left( {\alpha _1 + 2\beta _1} \right)U_3}}{{\sqrt 3 [\left( {\alpha _1\beta _2 + \alpha _2\beta _1} \right)U_1 - \alpha _2U_2 - \alpha _1U_3]}}} \right\}} \end{array}} \right.$$

### Data acquisition and analytics

The data of micro flow sensors, tri-axis accelerometer and tri-axis gyroscope in the wearable device are collected by an integrated MCU at a sampling frequency of 1000 Hz and are filtered in a MCU. The filtered data are transmitted wirelessly to a host computer through a Bluetooth at a frequency of 100 Hz and used to implement data fusion to figure out three-dimensional motion velocity, acceleration and attitude in real time. The reference motion data acquired by VICON system is collected by the host computer as well. A trigger pulse is used to synchronize the data transmission of our device and VICON system. The intra-limb coordination model is trained off line. And the trained intra-limb coordination model is used to determine thigh motion from shank motion in real time.

### Neural network training

The neural network model representing intra-limb coordination relationship of human lower limb is trained for each subject by using training datasets detected by optical VICON system. The training datasets are collected from a training experiment when the subject is walking and running on a treadmill with a velocity increasing from 0 to 10 km/h at an interval of 1 km/h. The shank motion data are used as the inputs and the thigh motion data are used as outputs of the neural network. The model training is carried out using iterative least square method in MATLAB software. The number of the hidden neurons is optimized by minimizing the RMSE of thigh attitude angles (*θ*_t_ and *γ*_t_) using least square method. The RMSE of *θ*_t_ and *γ*_t_ changing with the number of neurons is shown in Supplementary Fig. 11. The RMSE decreases with neuron number and gradually reaches to steady state. And the RMSE keeps nearly constant with more than 30 hidden neurons. Thus the three-layer neural network is optimized to have 30 hidden neurons. The trained intra-limb coordination model is validated by determining the thigh motion from the shank motion data detected by our device worn on the subject who conducts walking and running experiments similar with the training experiment. The motion data measured by our device are compared with that of the optical VICON system as shown in Fig. 7 and Supplementary Fig. 1 to Fig. 4. Four subjects with different ages including one patient suffering from mild meniscus injury participate in lower limb motion capture experiment, whose information is summarized in Supplementary Table 2. Three repeated validation experiments are conducted for each subject.

Experiments performed in studies involving human participants are approved by the Institution Review Board of Tsinghua University (No. 20180009). And informed consent is obtained from the human subjects to use the image and conduct the experiments described in this paper.

## Supplementary information

Supplementary Information

Supplementary Movie 1

Supplementary Movie 2

Supplementary Movie 3

Description of Additional Supplementary Files

## Data Availability

The data that support the findings of this study are available from the corresponding author upon reasonable request.

## Code availability

The codes that support the findings of this study are available from the corresponding author upon reasonable request.
