# Supplementary material for: A wearable motion capture device able to detect dynamic motion of human limbs
Source: Nat Commun. 2020 Nov 5;11:5615. doi: 10.1038/s41467-020-19424-2 (PMC7645594; doi:10.1038/s41467-020-19424-2)
Supplement: Supplementary file 1 — Supplementary Information [file 41467_2020_19424_MOESM1_ESM.pdf]

Supplementary Information for

A wearable motion capture device able to detect dynamic motion of human limbs

Liu et al.

Correspondence to: zr_gloria@mail.tsinghua.edu.cn

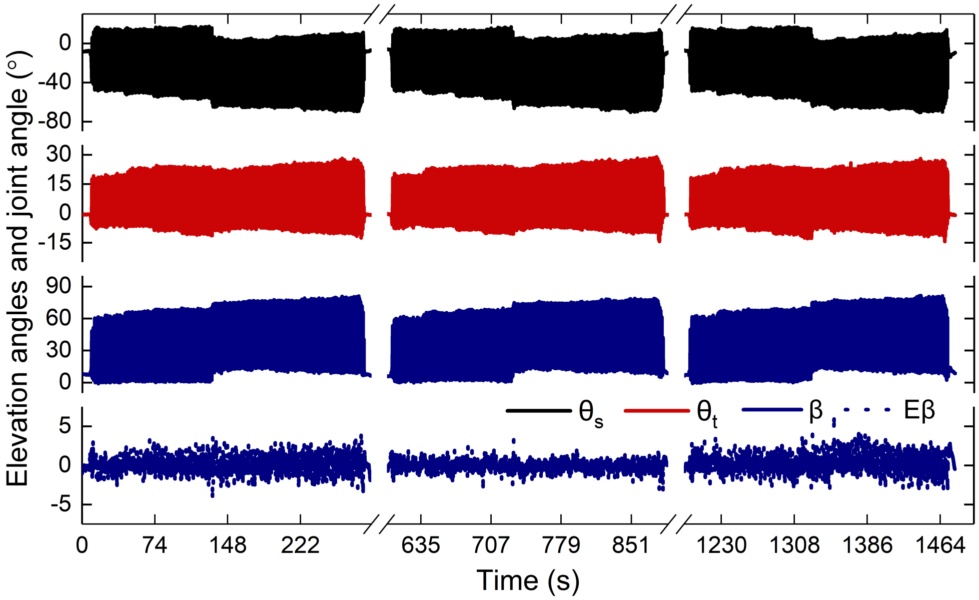

Supplementary Fig. 1| (Subject 1) Results of lower limb motion capture in the sagittal plane by using single device worn on the shank and determining the thigh motion from the shank motion by the trained neural network model of intra-limb coordination. The pitch angles of thigh θ_t and shank θ_s are used to represent the corresponding elevation angles in the sagittal plane respectively. The joint angle of knee β in the sagittal plane is derived by subtracting θ_s from θ_t. The error of β (Eβ) represents the estimation performance of both θ_t and β, including the measurement error of the wearable device and the model error of the neural network. The subject takes about 5-mins rest between three repeated experiments.

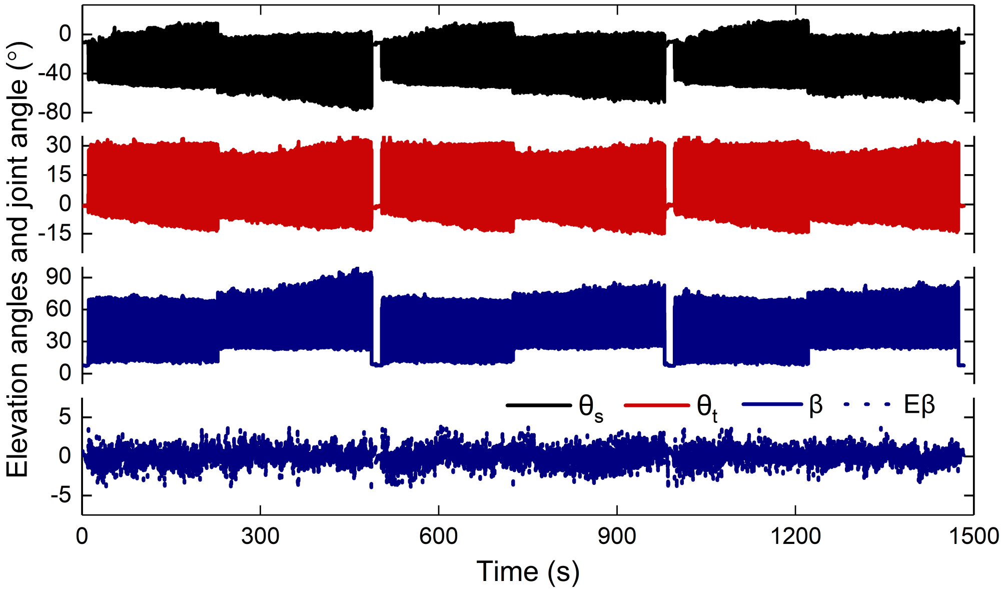

Supplementary Fig. 2| (Subject 2) Results of lower limb motion capture in the sagittal plane by using single device worn on the shank and determining the thigh motion from the shank motion by the trained neural network model of intra-limb coordination. The pitch angles of thigh θ_t and shank θ_s are used to represent the corresponding elevation angles in the sagittal plane respectively. The joint angle of knee β in the sagittal plane is derived by subtracting θ_s from θ_t. The error of β (Eβ) represents the estimation performance of both θ_t and β, including the measurement error of the wearable device and the model error of the neural network. Three repeated experiments are conducted continuously.

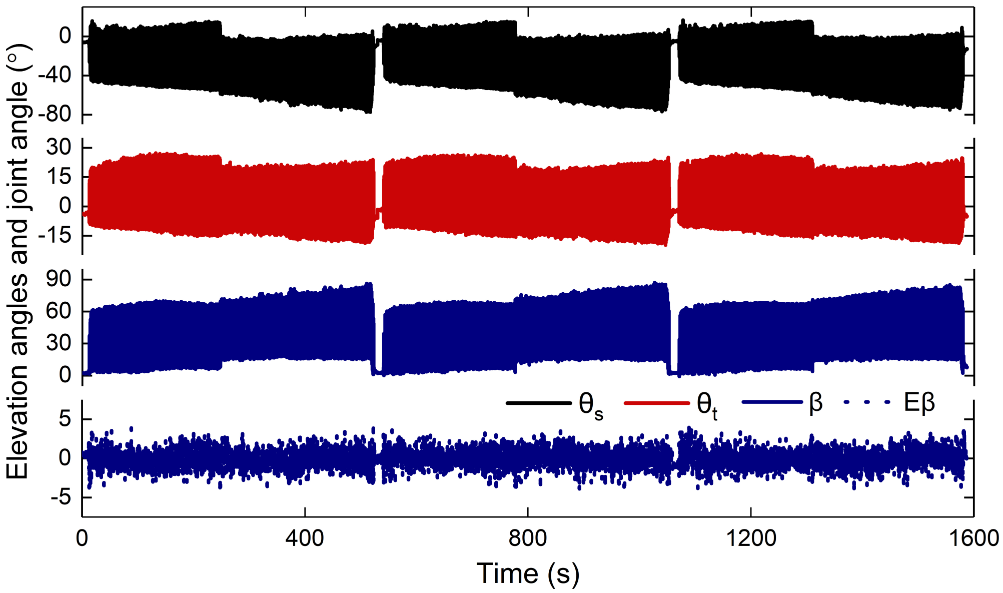

Supplementary Fig. 3| (Subject 3) Results of lower limb motion capture in the sagittal plane by using single device worn on the shank and determining the thigh motion from the shank motion by the trained neural network model of intra-limb coordination. The pitch angles of thigh θ_t and shank θ_s are used to represent the corresponding elevation angles in the sagittal plane respectively. The joint angle of knee β in the sagittal plane is derived by subtracting θ_s from θ_t. The error of β (Eβ) represents the estimation performance of both θ_t and β, including the measurement error of the wearable device and the model error of the neural network. Three repeated experiments are conducted continuously.

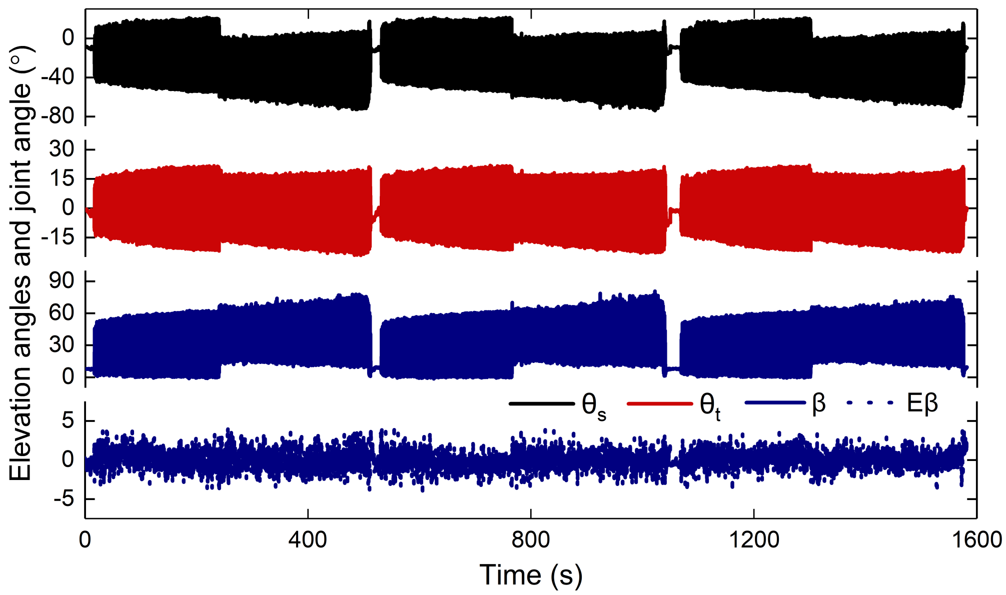

Supplementary Fig. 4| (Subject 4) Results of lower limb motion capture in the sagittal plane by using single device worn on the shank and determining the thigh motion from the shank motion by the trained neural network model of intra-limb coordination. The pitch angles of thigh θ_t and shank θ_s are used to represent the corresponding elevation angles in the sagittal plane respectively. The joint angle of knee β in the sagittal plane is derived by subtracting θ_s from θ_t. The error of β (Eβ) represents the estimation performance of both θ_t and β, including the measurement error of the wearable device and the model error of the neural network. Three repeated experiments are conducted continuously.

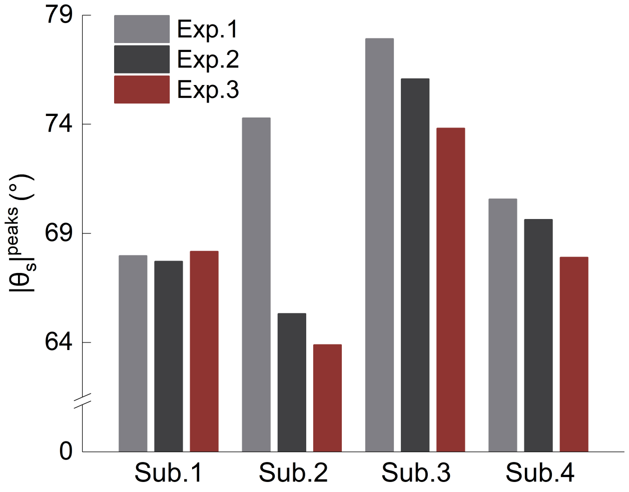

Supplementary Fig. 5| Maximum deflection of shank. Deflection of shank is the angle between the shank and the vertical and is represented by $|\theta_s|$ here. Maximum deflection of shank reveals the ability of lifting one's heel and can be used as an indicator of fatigue. For each subject, the maximum deflection of shank $|\theta_s|^{peaks}$ in each repeated experiment of lower limb motion capture is evaluated by averaging peak values of $|\theta_s|$ during the last 25 s running on a treadmill at velocity 10 km/h.

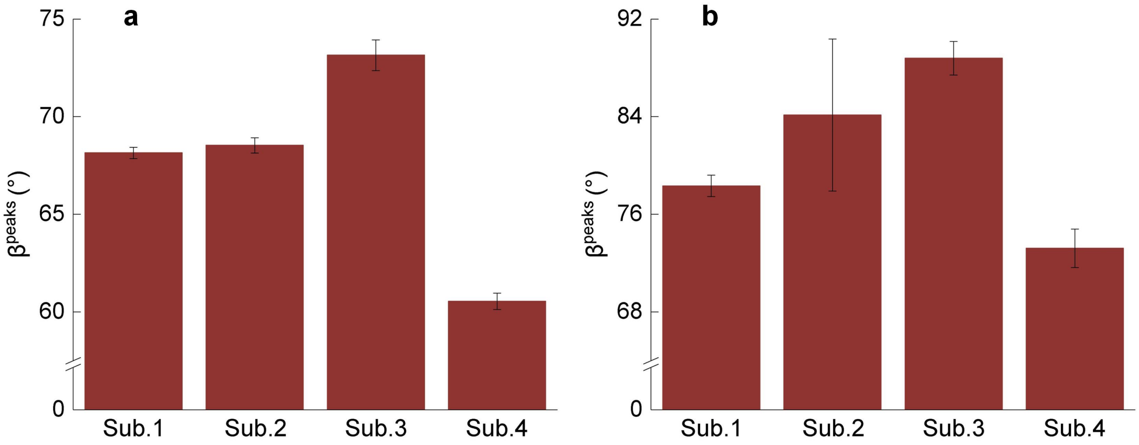

Supplementary Fig. 6| Comparison between knee flexions of subject with knee injury and those of healthy ones. a, Knee flexion ability of four subjects while walking on a treadmill at velocity 5 km/h. **b**, Knee flexion ability of four subjects while running on a treadmill at velocity 10 km/h. People with knee injury (Subject 4) suffer from weakened knee flexion ability and thus present smaller maximum knee angle than heathy people (Subject 1, 2, and 3) when walking and running due to hurt or pathological knee constraints. Maximum knee angle β^{peaks} is used as an indicator of knee flexion ability, which is evaluated by averaging peak values of β during the last 25 s walking at velocity 5 km/h and running at velocity 10 km/h in three repeated experiments of lower limb motion capture. Error bars here represent standard deviation (SD).

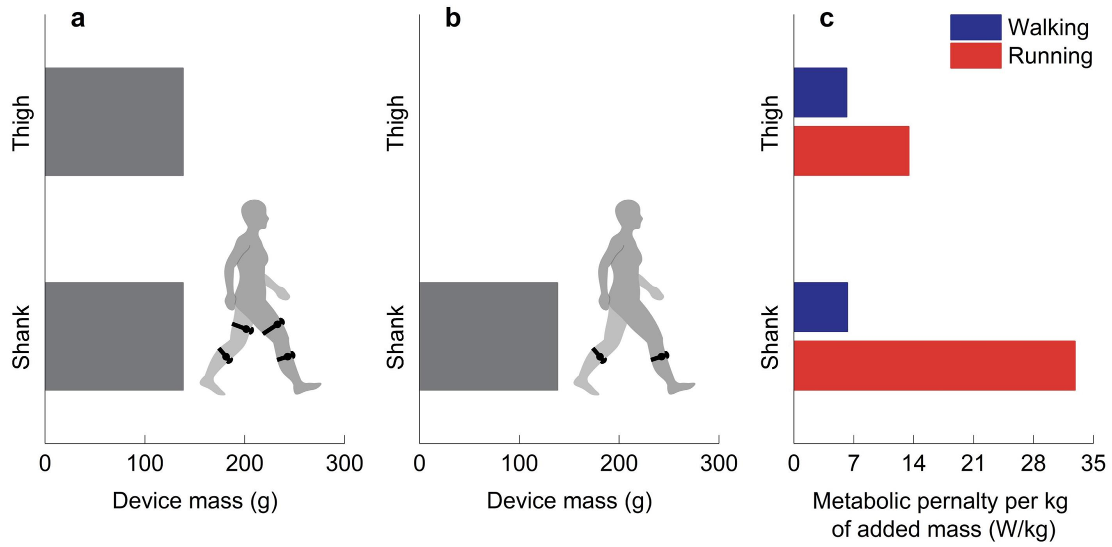

Supplementary Fig. 7| The complexity and metabolic penalty of lower limb motion capture by different methods. a-b, The number of motion capture device and corresponding bilateral added mass by using conventional motion capture method (**a**) and our proposed method (**b**). **c**, The estimated metabolic penalty per kilogram of added mass for each segment, based on coefficients from the literature [25,58,59]. The setup for lower limb motion capture by using different methods and the corresponding metabolic penalty are shown in Supplementary Table 3.

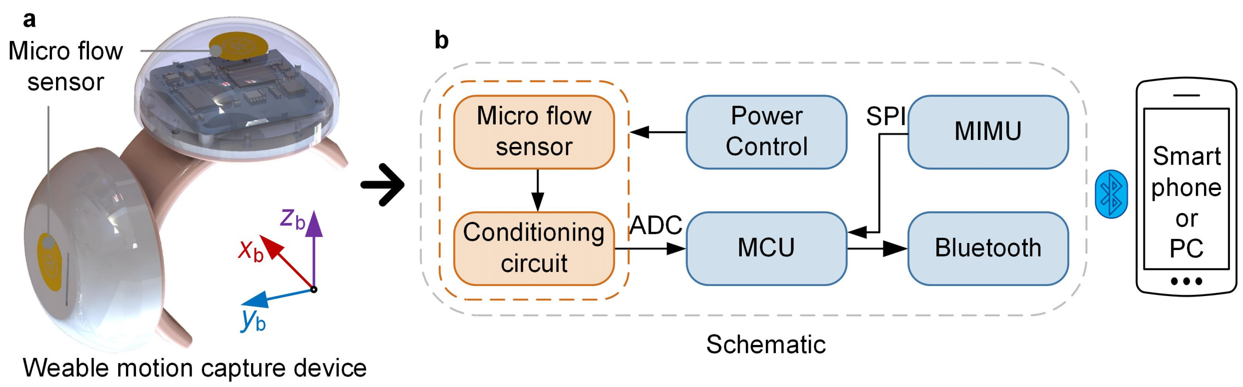

Supplementary Fig. 8| Configuration of the wearable device. a, The device using two orthogonally-placed micro flow sensors to measure tri-axis motion velocity. **b**, Micro flow sensors are integrated with a MIMU in the device, outputs of which are collected by a MCU. Bluetooth is used to wirelessly transmit the device data to smart phone or PC.

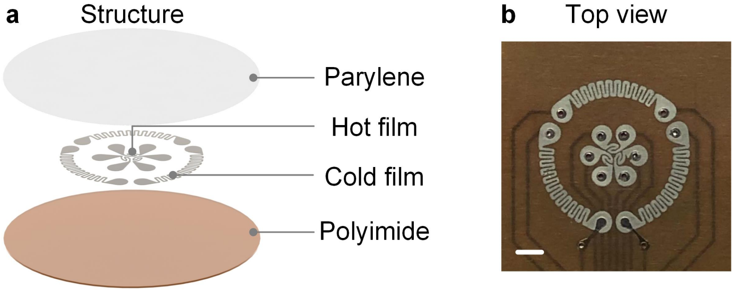

Supplementary Fig. 9| Structure of the micro flow sensor. a, Detailed structure of the micro flow sensor consists of polyimide substrate, platinum thermo-sensitive layer including three central platinum ribbons (denoted as hot films) and three circumambient platinum ribbons (denoted as cold films) all of which are sealed by a protective layer of parylene. **b**, Fabricated prototype of the micro flow sensor. The scale bar is 1mm.

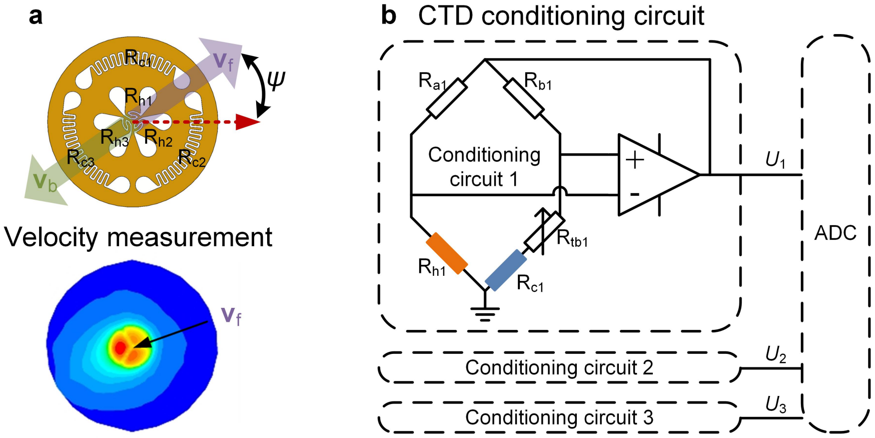

Supplementary Fig. 10| Principle of motion velocity measurement. a, A micro flow sensor measures two-dimensional motion velocity by detecting motion induced surface flow. **b**, The conditioning circuit is composed of three constant temperature difference (CTD) circuits. Using the CTD circuit of the first pair of hot film (denoted as R_{h1}) and cold film (denoted as R_{c1}) as an example, R_{h1} and R_{c1} are connected into a Wheatstone bridge with two resistors (R_{a1} and R_{b1}), the differential voltage is amplified and then fed back to the bridge to implement the CTD. The resistor R_{tb1} is used to regulate the temperature difference between hot film and ambient. The bridge top voltage is further converted to digital signal by an analog/digital converter (ADC) for sampling. Outputs of the three CTD circuit are used to figure out the planar motion velocity $\mathbf{v}_b$.

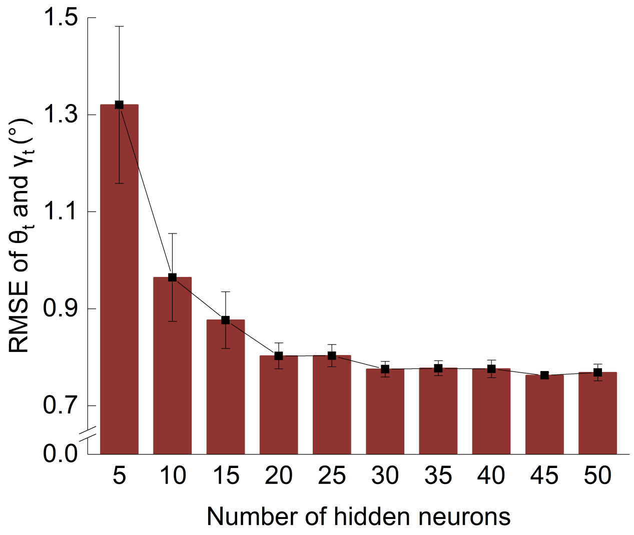

Supplementary Fig. 11| Optimization of hidden neurons. The RMSE of θ_t and γ_t is used as the performance index of hidden neuron optimization. The smaller the RMSE is, the better performance the network has. The RMSE decreases with neuron number and gradually reaches to steady state. The RMSE keeps nearly constant with more than 30 hidden neurons. Network training is conducted repeatedly three times with the same hidden neurons. Error bars here represent standard deviation (SD).

Limbs	Errors of attitude (º)	Inertial method	Our device
Forearm in boxing	ME of γ	-3.19	-0.30
	RMSE of γ	3.78	1.70
	ME of θ	-1.68	-0.09
	RMSE of θ	4.18	1.14
Shank in kicking	ME of γ	-1.15	0.47
	RMSE of γ	1.47	0.68
	ME of θ	-1.93	-0.06
	RMSE of θ	2.94	0.41

Supplementary Table 1| Experiment results of dynamic motion capture in strenuous exercises. ME is the mean error of the attitude angle, RMSE is the root-mean-square error of the attitude angle. ME is able to reflect the drift error of short-time motion capture. RMSE reflects the accuracy and stability of motion capture.

Sub. No.	Age (year)	High (cm)	Weight (kg)	Errors of knee angle (º)						
				Exp. No.	1		2		3	
				Error	Sagittal plane β	Coronal plane α	Sagittal plane β	Coronal plane α	Sagittal plane β	Coronal plane α
1	40	170	80	ME	0.15	-0.16	-0.05	0.10	0.31	-0.22
				RMSE	1.07	0.64	0.63	0.49	1.18	0.73
2	22	171	72	ME	0.03	-0.08	-0.02	-0.14	0.03	0.06
				RMSE	1.00	0.39	1.16	0.44	0.95	0.38
3	31	175	75	ME	-0.09	-0.21	0.11	0.22	-0.05	0.06
				RMSE	1.09	0.42	1.05	0.42	1.09	0.44
4*	28	175	65	ME	0.09	0.20	-0.12	0.66	0.12	-0.66
				RMSE	1.20	0.56	1.07	0.58	0.98	0.51

Supplementary Table 2| Measurement errors of knee joint angle in lower limb motion capture by using single device worn on the shank and determining the thigh motion from the shank motion by the trained neural network model of intra-limb coordination. The joint angle of knee β in the sagittal plane is derived by subtracting θ_s from θ_t. The joint angle of knee α in the coronal plane is derived by subtracting γ_s from γ_t. ME is the mean error of the joint angle, RMSE is the root-mean-square error of the joint angle. ME is able to reflect the drift error of joint angle measurement. RMSE reflects the accuracy and stability of joint angle measurement.

* Mild meniscus injury.

Method	Bilateral limbs	Device quantity	Bilateral added mass* (g)	Expected metabolic penalty (W)	
				Walking** at 5 km/h	Running*** at 10 km/h
Conventional inertial method	Thighs	2	138.2	0.85	1.86
	Shanks	2	138.2	0.87	4.54
	Total	4	276.4	1.72	6.40
Our method	Thighs				
	Shanks	2	138.2	0.87	4.54
	Total	2	138.2	0.87	4.54

Supplementary Table 3| The complexity and metabolic penalty of different methods of lower limb motion capture. The number of motion capture device, the corresponding bilateral added mass and estimated metabolic penalty by using the conventional method and our proposed method for lower limb motion capture of walking and running. The metabolic penalty of added mass for each segment is estimated using penalty coefficients obtained from the literature [25,58,59] which are linearly rescaled according to the velocities used in our research.

[*] The weight of our proposed motion capture device is 69.1 g.

[**] Metabolic penalty coefficients of thigh and shank [25,58] are c_t=6.180 W/kg and c_s=6.262 W/kg, respectively.

[***] Metabolic penalty coefficients of thigh and shank [25,59] are c_t=13.433 W/kg and c_s=32.856 W/kg, respectively.
