# Supplementary material for: A wearable motion capture device able to detect dynamic motion of human limbs
Source: Nat Commun. 2020 Nov 5;11:5615. doi: 10.1038/s41467-020-19424-2 (PMC7645594; doi:10.1038/s41467-020-19424-2)
Supplement: Supplementary file 5 — Description of Additional Supplementary Files [file 41467_2020_19424_MOESM5_ESM.pdf]

Title: Supplementary Movie 1
Description: Boxing motion capture of forearm wearing our device.

Title: Supplementary Movie 2
Description: Kicking motion capture of shank wearing our device.

Title: Supplementary Movie 3
Description: Motion capture of lower limb by wearing our device on the shank.
